# Understanding and treating menstruation associated sickle cell pain

**DOI:** 10.1186/s40834-025-00361-8

**Published:** 2025-04-03

**Authors:** Halimat Olaniyan, Bria Carrithers, Layla Van Doren

**Affiliations:** 1 Department of Clinical Pathology and Laboratory Medicine, School of Medicine, Indiana University, Bloomington, USA; 2 Section of Hematology, Department of Medicine, Yale School of Medicine, 333 Cedar Street Room WW201, New Haven, CT 06520 USA

**Keywords:** Sickle cell disease, Contraception, Pain, Menstruation

## Abstract

Sickle cell disease (SCD) is a chronic inflammatory condition characterized by hemoglobin polymerization that precipitates recurrent vaso-occlusion, endothelial dysfunction, and multi-organ damage. Menstruation in persons with SCD presents a unique challenge due to blood loss and its ability to exacerbate SCD pain. This interaction between SCD-related vascular stress and menstruation-induced inflammation amplifies the risk of acute pain episodes during menstruation. In this manuscript, we explore the intersection of SCD and menstruation, emphasizing the role of hormonal therapy in managing menstruation-associated acute SCD pain. Progestin-only therapies, such as depot medroxyprogesterone acetate (DMPA) and levonorgestrel intrauterine devices (LNG-IUDs), are particularly effective in reducing menstrual blood loss. Data suggests DMPA mitigates acute SCD pain episodes around menstruation with minimal thrombotic risk in persons with SCD. Despite their effectiveness in menstrual regulation, combined hormonal contraceptives (CHCs) pose a significant concern due to their potential to exacerbate the hypercoagulable state in individuals with SCD. We highlight the importance of comprehensive care and collaboration between gynecologists and hematologists to optimize the management of menstruation-associated SCD pain.

## Background

Sickle cell disease (SCD) is a complex inflammatory condition marked by hemoglobin polymerization that leads to chronic and progressive damage affecting all organ systems, including the reproductive organs [1]. The pathophysiology of SCD involves activation of both the innate and adaptive immune responses, chronic inflammation, and endotheliopathy, contributing to a range of long-term complications, such as acute on chronic pain and increased thrombosis risk [2]. Acute pain remains the leading cause for presentation to the emergency department and hospitalization in persons with SCD [3]. Menstruation poses unique challenges for persons with SCD as hormonal fluctuations and menstrual blood loss can trigger or exacerbate SCD pain.

A key consequence of intravascular hemolysis is the release of free hemoglobin, which scavenges nitric oxide (NO), a vasodilator and regulator of endothelial function. The resultant decrease in NO bioavailability leads to endothelial injury, leukocyte adhesion, increased platelet aggregation, and ischemia [1]. Additionally, hormonal fluctuations during menstruation have shown to contribute to inflammation during menstruation, amplifying leukocyte adhesion, endothelial vasoconstriction, and promoting microvascular occlusion within the uterine cavity [4]. These events might perpetuate the pro-inflammatory state in persons with SCD, leading to menstruation-associated SCD pain.

Women, girls, and menstruating persons with SCD experience a higher prevalence of dysmenorrhea, heavier menstrual bleeding, and menstruation-associated acute SCD pain, resulting in significant impacts on quality of life and healthcare utilization [5, 6]. Hormonal therapy offers a promising approach to alleviate dysmenorrhea, reduce blood loss, and reduce menstruation-associated SCD pain. However, choosing the most appropriate method is nuanced. Due to the thrombophilia of SCD, progestin-only options, such as depot medroxyprogesterone (DMPA) and the levonorgestrel (LNG) intrauterine device (IUD), are generally favored. However, an individualized approach to hormonal therapy is essential to balance menstrual and vascular health with the patient’s personal preferences and reproductive goals. Collaboration between the hematologist providing SCD care and gynecologist is crucial to ensure comprehensive care tailored to the needs of persons with SCD.

## Menstruation-associated acute SCD pain

A relationship between menstruation and SCD pain has been well established [5, 7, 8]. Women, girls, and menstruating persons with SCD are more likely to endure acute SCD pain during their reproductive years, with higher rates of pain overall compared to men with SCD. In a multinational study, 39% of women reported menstruation-associated pain crises in their lifetime. These women were significantly more likely to be hospitalized compared with those who did not report menstruation-associated SCD pain (mean 1.70 vs. 0.67, *p* = 0.0005). Women reporting menstruation-associated SCD pain within a six-month period also experienced increased hospitalizations compared with those who did not (mean 1.71 vs. 0.75, *p* = 0.0016). 40% of women reported at least four menstruation-associated SCD pain episodes over a six-month period [9]. Those affected often describe their SCD pain as distinct from dysmenorrhea and consistent across menstrual cycles [8]. A prospective study investigating the cyclical nature of menstruation-associated SCD pain in 213 participants reported 61.5% of participants experienced pain only during menstruation, while 34.5% of participants’ SCD pain began during the premenstrual phase (between day 21 and 28) and continued throughout menstruation [10]. Over 30% of participants experienced SCD pain with every cycle over the six-month study period [9]. Notably, menstrual flow is reported to be longer and heavier in persons with SCD compared to those without, lasting 4.9 days compared to control group lasting 4.3 (*p* < 0.006) [10]. Menstruation-associated SCD pain demonstrates the complex pain paradigm in SCD, as increased hospitalizations highlight healthcare utilization, emotional well-being, and significant impacts on quality of life. This underscores the need for effective and tailored management strategies to mitigate health inequity amongst women, girls, and those who menstruate with SCD.

The use of hormonal therapy methods offers promising benefits for menstruation in persons with SCD. This includes improvement in menstrual blood loss, management of dysmenorrhea, and mitigation of menstruation-associated SCD pain [6]. Menstrual suppression, but not necessarily ovulation, can be achieved with a success rate of 50–75% with multiple hormonal therapies [11]. Despite the potential benefits of hormonal therapy, previous studies have reported low knowledge of hormonal therapy efficacy and use of long-acting reversible contraceptives (LARC) in persons with SCD [12, 13]. Yet, the effects of hormonal therapy on SCD symptoms remains a priority for patients, underscoring the need for providing person-centered informed care with the use of hormonal therapy for management of menstruation-associated SCD pain.

Initiation of discussions with patients regarding contraceptive preferences provide an opportunity to highlight inequities that impact sexual and reproductive health (SRH) for patients with SCD. For example, in a national study evaluating pediatric hematology providers’ contraceptive practices for female adolescents and young adults with SCD, most providers reported contraception counseling with patients, yet only 41.8% report prescribing contraception [14]. Key motivators to contraceptive counseling included patient request and disclosure of sexual activity, but not menstruation-associated SCD pain. Individuals with SCD desire to have conversations regarding their reproductive health with their SCD provider [15]. As patients with SCD do not always recognize menstruation as a cause for SCD pain, effective discussions and management of both menstrual bleeding and menstruation-associated SCD pain is essential for comprehensive SRH care in individuals with SCD.

## Hormonal therapy to manage menstruation-associated SCD pain

### Progestin-containing therapy and SCD

Progestin only therapies are the most frequently used hormonal therapies in SCD, as they have been associated with a decrease in menstruation-associated SCD pain [6]. Depot medroxyprogesterone (DMPA) and the levonorgestrel (LNG) intrauterine device (IUD) are the most commonly preferred by persons with SCD and their providers, with DMPA the best studied contraception in SCD [13, 16]. In a systematic review of eight studies evaluating the use of progestin-only therapies in over 300 individuals with SCD, users experienced fewer and less intense acute SCD pain episodes compared with non-users [17]. No adverse sickle cell related events have been reported using any progestin-only method, leading most SCD providers to recommend these as first line for persons with SCD [16, 18]. However, DMPA has been shown to have a slight increased risk of venous thrombosis in the general population compared with non-use [19], leading the 2024 United States (U.S.) Medical Eligibility Criteria (MEC) for contraceptive use to recategorize it as category 2/3 in persons with SCD, noting that **“**it should be assessed according to the severity of SCD and risk of thrombosis.” A notable effect that is unique to DMPA is bone mineral density loss over time which has shown to resolve at some point after cessation. This effect of DMPA has not specifically been studied in persons with SCD, who are already at risk of osteopenia and osteoporosis by virtue of their condition [20]. Other factors such as route of administration and non-SCD related side effects may impact the use of various progestine only therapies (Table 1).

**Table 1:** Contraceptives for the management of menstruation associated SCD pain

Therapy	Mechanism of action*	Route of administration	Clinical evidence in SCD	Comments
**Progestin-only therapies** Primary mechanism of pregnancy prevention is thickening of cervical mucus.			Legardy, et al. 2006 [17] Yoong et al. 1999 [10]	No SCD adverse events, hematologic or other biochemical adverse parameters, and no thrombosis have been reported with any of the progestin-only methods.
**Depot medroxyprogesterone (DMPA)**	LARC Suppress ovulation	Intramuscular injection every 3 months	de Abood et al., 1997 [7] De Ceulaer et al., 1982 [34] Howard et al., 1993 [35]	• Associated with a decrease in menstruation associated SCD pain and frequency. • Side effects: unscheduled bleeding, weight gain, and bone mineral density loss over time. • Higher risk of VTE in the general population, compared with no use **CDC MEC 2024 Category 2/3** [34]**
**Levonorgestrel (LNG) IUD**	LARC Suppress Menstruation & inconsistently suppress ovulation	Intrauterine device every 5–8 years	Howard et al., 1993 [35]	• High rates of amenorrhea and contraception are achieved with the LNG IUD. **CDC MEC 2024 Category 1**
**Etonogestrel (ENG) implant**	LARC Suppress ovulation for 3 years and prevents pregnancy for 5 years	Subcutaneous implant every 5 years	Nascimento et al.1998 [21] Ladipo et al. 1993 [22]	• Nomegestrel acetate resulted in a decline in headaches, body weakness, and limb pain in persons with SCD. • Studies with ENG implant and SCD are ongoing. • Unscheduled bleeding and spotting can be unpredictable leading to a higher rate of discontinuation compared with other LARCs. **CDC MEC 2024 Category 1**
**Progestin only pill** **(Norethindrone**,** Drospirenone)**	Inconsistent suppression of ovulation	Oral, daily	Howard et al. 1993 [35]	• Small therapeutic window to maintain the full contraceptive benefits. • Can result in intermittent bleeding. **CDC MEC 2024 Category 1**
**Other hormonal and non-hormonal therapies**				
**Combined hormonal contraceptive (CHC)**	Suppress ovulation and produce a regular consistent bleeding pattern	Oral pill, transdermal patch, and vaginal ring	de Abood et al. 1997 [7] Howard et al. 1993 [35] Yoong et al. 1999 [10]	• Few studies evaluate have evaluated CHCs in SCD. • CHCs are associated with a relative increased risk for VTE compared to non-users in the general population. • SCD is a high-risk thrombophilia and CHCs are generally avoided in this population. *A thoughtful discussion and shared decision making weighing the risks and benefits should be had when considering CHC use in SCD. **CDC MEC 2024 Category 4**
**Copper (Cu) IUDs**	Copper ions prevent sperm mobilization	Intrauterine device every 10 years	N/A	• Can lead to worsening menstrual cramps and increased bleeding, so it is generally not ideal for use in persons with SCD. **CDC MEC 2024 Category 2** (Concern exists about an increased risk of blood loss with Cu-IUDs)

CHC, combined hormonal contraceptive; POP, progestin only pill; DMPA, depot medroxyprogesterone; ENG Implant, Subdermal Etonogestrel Implant; CU-IUD, copper intrauterine device; LNG-IUD, levonorgestrel intrauterine device.

U.S. MEC Category 1 = A condition for which there is no restriction for the use of the contraceptive method.

U.S. MEC Category 2 = A condition for which the advantages of using the method generally outweigh the theoretical or proven risks.

U.S. MEC Category 3 = A condition for which the theoretical or proven risks usually outweigh the advantages of using the method.

U.S. MEC Category 4 = A condition that represents an unacceptable health risk if the contraceptive method is used.

*Mechanism of action for menstrual suppression and prevention of pregnancy.

** It should be assessed according to the severity of SCD and risk of thrombosis.

The long acting reversible contraceptives (LARCs) include subdermal implants and IUDs. The nomegestrel acetate subdermal implant resulted in a decline in headaches, body weakness and limb pain in persons with SCD [21]. The only other study to evaluate the subdermal LNG implant in persons with SCD did not show any change in hematologic or biochemical parameters [22]. Pain was not an endpoint. Currently, the etonogestrel (ENG) implantable progestin, Nexplanon^®^ (Merck, United States) is the only FDA approved implantable device. There is ongoing work to measure its impact in SCD (ClinicalTrials.gov Identifier NCT05730205). The LNG IUDs are other LARCs that have been well established to be safe and effective in persons without SCD, including adolescents and young adults (AYA) [23]. Although the LNG IUD has not been specifically studied in persons with SCD, it is the second most commonly preferred hormonal contraception by patients [13]. The benefit of the LNG IUD is its route of administration, typical use failure rate of < 1%, and high rate of amenorrhea of approximately 50% at one year, and 80% of patients reporting improvement in menstrual blood loss [24]. LNG IUD’s do not consistently suppress ovulation, and this should be taken into consideration when suppression of ovulation is desired, since it is unkown if menstrual suppression, ovulation suppression, or both are necessary to alleviate menstruation-associated SCD pain. DMPA and the ENG subdermal implant are progestin only contraceptives that consistently suppress ovulation. The copper IUD is a non-hormonal LARC option, but has been associated with increased dysmenorrhea and heavier menses and is therefore not ideal in persons with SCD [25].

### Estrogen-containing therapy and SCD

Combined hormonal contraceptives (CHCs) suppress ovulation and produce a regular and consistent bleeding pattern. Since their introduction, oral CHCs have been associated with a relative increased thrombosis risk; however, the absolute risk for persons without SCD remains low [26]. The estrogen component of CHCs contributes to a pro-thrombotic state, thought to be the result of alterations in the hemostatic and fibrinolytic pathways [27]. Variations in the dose of estrogen, type of progestin, and route of administration influence the thrombotic risk [23]. For non-oral estrogen-based therapies, such as the transdermal patch and vaginal ring, the associated thrombosis risk remains less clearly defined.

Separately, SCD pathophysiology is associated with a considerable pro-thrombotic state. By age 40, approximately 11.2% of persons with SCD experience a clinically significant venous thromboembolism (VTE) [28]. Homozygous SS and sickle cell beta-thalassemia (S$\:\beta\:$^0^) genotypes are associated with a higher risk of both VTE and stroke [29]. Furthermore, silent cerebral infarcts affect approximately 39% of persons with SCD by age 18 and over 50% by age 30 [30]. A history of a stroke in the general population presents an unacceptable health risk for CHC use [31]. Therefore, many providers consider CHC use in persons with SCD to be an unacceptable health risk due to the high rate of cerebral infarts. The impact of CHCs on sickle cell pain remains uncertain, however. One study reported a reduction in menstruation-related SCD pain among CHC users compared to those using no contraception, albeit at a lower rate than those using DMPA [7]. A systematic review of the safety of hormonal contraceptives in SCD found that neither progestin-only methods nor CHCs increased the frequency of pain crises or other adverse outcomes [18]. Disease-modifying therapies (DMT), such as hydroxyurea, and chronic red blood cell exchange, may reduce the hypercoagulable state of SCD [32].

In a 2021 survey study of 78 pediatric SCD physicians and advanced practice providers, 23% agreed or strongly agreed that combined hormonal contraceptives (CHCs) should never be used in adolescents and young adults with SCD. 51% agreed or strongly agreed that CHCs can be considered if “no other risk factors are present [16].” The 2024 U.S. MEC for conctraceptive use lists the use of CHCs as category 4 in persons with SCD, indicating an unacceptable health risk [31]. This is a change from the 2016 U.S. MEC for contraceptive use, which listed CHCs in SCD as category 2 [33]. Variation in recommendations from national organizations regarding this topic have likely contributed to variation in practice regarding the use of CHCs in this population. Depending on genotype, DMT, and anticoagulation use, CHCs might be a viable option for those seeking better overall reduction in menstruation associated symptoms whom progestin-only methods have been ineffective or not desired.

## Conclusion

The intersection of menstruation and SCD presents unique challenges due to vascular stress induced by both conditions and exacerbation of SCD pain. We provide a guide for the use of hormonal therapies in mitigating menstruation-associated SCD pain. Effectively managing menstrual bleeding and menstruation-associated SCD pain is essential for comprehensive SCD care. Progestin-only therapies are shown to be safe and effective options that provide symptom relief while minimizing thrombotic risks. If suppression of ovulation is desired, DMPA or ENG implantable devices may be the ideal initial choices, noting that irregular bleeding may occur. Meanwhile, CHCs present significant thrombotic concerns in SCD but may still hold relevance in certain cases depending on genotype, disease-modifying therapy, anticoagulation use, or where menstrual symptom management is prioritized.

The limited knowledge and utilization of hormonal therapies among persons with SCD highlights a pressing need for enhanced educational efforts targeting both patients and providers to support informed decision-making in an effort to decrease symptom burden while posing minimal risks to patients. A collaborative approach between hematologists and gynecologists is essential to provide comprehensive SRH care in SCD. Given the inherently personal nature of contraception choices, the diversity of contraceptive methods necessitates tailoring contraceptive counseling to the patient’s needs. Whether targeting symptom relief, ovulation suppression, or other reproductive goals, this ensures alignment with each individual’s health profile and preferences, advancing both health equity and reproductive justice for persons with SCD.

## Data Availability

No datasets were generated or analysed during the current study.

## Abbreviations

SCD: Sickle cell disease
SRH: Sexual reproductive health
DMPA: Depot medroxyprogesterone
CHC: Combined hormonal contraceptive
CU-IUD: Copper intrauterine device
ENG: Etonogestrel
LNG: Levonorgestrel
AYA: Adolescents and young adults
VTE: Venous thromboembolism
CDC: Centers for Disease Control and Prevention
MEC: Medical Eligibility Criteria
